# Mapping the Locations of Medical Specialists in the Ministry of Health’s Hospitals in Malaysia by Specialty, Subspecialty and Area of Interest

**DOI:** 10.21315/mjms2024.31.3.1

**Published:** 2024-06-27

**Authors:** Hirman Ismail, Mohamed Hirman Abdullah, Sabrizan Osman, Faarhana Che Arshad, Siti Norsyazwanis Jalaluddin, Nur Fadzilah Osman, Nor Akmal Hakim Kamarulzaman, Badiuzzaman Abd Kadir

**Affiliations:** Medical Development Division, Ministry of Health Malaysia, Putrajaya, Malaysia

**Keywords:** healthcare system, medical specialty, medical subspecialty, specialist training, Ministry of Health, human resource for health

## Abstract

Mapping the distribution of medical specialists in the Ministry of Health (MOH) Malaysia facilities is expected to be more complex as the demand for specialty and subspecialty services increases in the future. A more robust and definitive gap analysis is needed to facilitate planning and resource allocation. The Medical Development Division developed a master list of framework of specialties, subspecialties and areas of interest, and Specialist Database Module in the Medical Programme Information System (MPIS) as tools to facilitate mapping of services. Relational database of specialists’ location, facilities, workload, population profile and other relevant parameters were developed to provide data visualisation in specific dashboard. Needs versus supply ratio is proposed as one of parameters to visualise specialised medical services distribution by geographical localities.

## Introduction

There are 149 hospitals in the Ministry of Health (MOH) Malaysia of which 14 are state hospitals and 29 are major specialist hospitals, mostly offering multiple wide range of specialty and subspecialty services. There are 26 minor specialist hospitals offering mainly basic specialty services, while the remaining 69 are non-specialist hospitals. On top of that 11 hospitals are considered special medical institutions offering specific services such as psychiatric hospital, women and children’s hospitals and a special hospital for cancer treatment. Total beds capacity of all these hospitals is 45,848 beds. There are at least 14 new hospitals under construction and 26 projects for extension of existing hospitals, which are expected to increase hospitals capacity by at least 5,600 beds. In hospitals, there were approximately 2.5 million admissions, 14.5 million clinic outpatient attendances, 300,000 deliveries and 5.6 million emergency department attendances annually, making the MOH Malaysia the biggest healthcare provider in the country. Demand for specialised and subspecialised services in the future is expected to be higher as disease burden increases and socio-economic status changes in various localities in the country. The existing facilities shall be equipped and strengthened from time to time and more facilities would be developed to keep up with all the demand. It is therefore important to ensure the existing locations and distribution of medical specialists are properly mapped to identify gaps and subsequently measures can be taken to address the gaps accordingly and specifically to a locality. The MOH Malaysia produces around 800–900 specialists per year and 400 subspecialists per year. The distribution of these specialists and reallocation of the existing specialists is expected to be more complex as we anticipate various challenges such as posts availability and facilities readiness. A more robust system and mechanism is needed to map the dynamic location and distribution of medical specialists in various specialty and subspecialty areas. This is consistent with strategic recommendations of the World Health Organization (WHO) to monitor and manage distribution of human resource for health in the public sector, coupled with other strategies including education, regulation, incentives and other personal or professional support (1).

### Taxonomy of Medical Specialties

The Medical Development Division, MOH Malaysia has reorganised list of specialty and subspecialty areas and areas of interest. This is to assist in planning of expansion of specialised services within all 149 hospitals under the MOH Malaysia. Reorganising these diverse clinical areas is also intended to facilitate data collection, mapping of clinical services and planning of specialty and subspecialty training. According to the Malaysian Medical Council (MMC), ‘specialty’ in medicine is a branch of medical practice and is defined as an area of medicine with a broad-based body of knowledge that is relevant in both community and tertiary settings and is a foundation for additional competencies (2). ‘Subspecialty’ is a narrow field within a specialty; an area of medicine with a more focused or advanced scope that builds upon the broad-based body of knowledge defined in a parent specialty (2). Subspecialty training means a structured, at least 3 years, on-the-job advance training programme conducted by the MOH Malaysia for medical specialists, on clinical areas identified by the MOH Malaysia as subspecialty areas and/or recognised by the MMC as subspecialty areas. The 3-year duration of subspecialty training is an administrative definition, and it was also stipulated in the existing MMC procedure to register specialist in subspecialty areas (3). It is important to note that there are 113 subspecialty areas available in the MOH Malaysia of which 65 areas are listed under the National Specialist Register (NSR) under MMC, while the rest remained unlisted. NSR is a central register of all specialists in Malaysia in both public and private sectors, maintained by the MMC. Area of interest training on the other hand is also defined administratively as a structured, 1 year–2 years, on-the-job advance training programme conducted by the MOH Malaysia for medical specialists, on clinical areas identified by the MOH Malaysia as an area of interest. Area of interest has not been defined by the MMC and there are 107 areas listed under the MOH Malaysia training programme. ‘Specialty’ and ‘subspecialty’ areas were not explicitly defined in the Medical Act 1971 [Act 50]. List of registrable specialty and subspecialty areas are published on the MMC website. Inclusion of new speciality and subspecialty areas for registration under the NSR is guided through a guideline of the MMC (2).

These clinical areas were reorganised under a master list and are categorised into seven broad categories of specialty areas: i) medical-related, ii) surgical-and-anaesthesia-related, iii) mother-and-child-related, iv) community-related, v) pathology-and-lab-related, vi) radiology-related and vii) dental-related. It is important to include dental specialties into the listing as some of dental specialist services are also provided in the hospitals. In the master list, all subspecialty areas, regardless of whether there are listed or not listed under the NSR, and all areas of interest under each basic specialties are catalogued and given specific code numbers. The list is very dynamic and is maintained by the Medical Development Division through governance by a committee on subspecialty training. The committee, which consist of senior consultants, would study and approve any proposal on development of new areas. The master list was first approved in March 2022 (Appendix).

### Business Intelligence Process of Specialists Database Module in the Medical Programme Information System

The Medical Development Division developed a Specialist Database Module in the Medical Programme Information System or MPIS, following the reorganisation of list of specialty and subspecialty areas under the master list. MPIS is an online cloud-native web application developed by the Hospital Services Management Unit (under the Medical Development Division) in the year 2020 during the COVID-19 pandemic era, initially intended mainly to monitor hospital capacity and preparedness during the pandemic such as critical and non-critical beds occupancy, low-risk quarantine centres beds occupancy and COVID-19 clinical management. The MPIS has been extended to include other modules, such as COVID-19 mortality module, medical equipment application system and inpatient clinical management module. MPIS, previously known as Crisis Preparedness Response Centre (CPRC) Hospital System, is one of important contributors to data repository during the pandemic to facilitate the government’s informed decision-making during the crisis (4). Data collected through MPIS are all stored and secured in the public sector data centre or Pusat Data Sektor Awam (PDSA) hosted by the government’s Malaysian Administrative Modernisation and Management Planning Unit (MAMPU), also known as MyGovCloud@PDSA.

**Figure 1:**
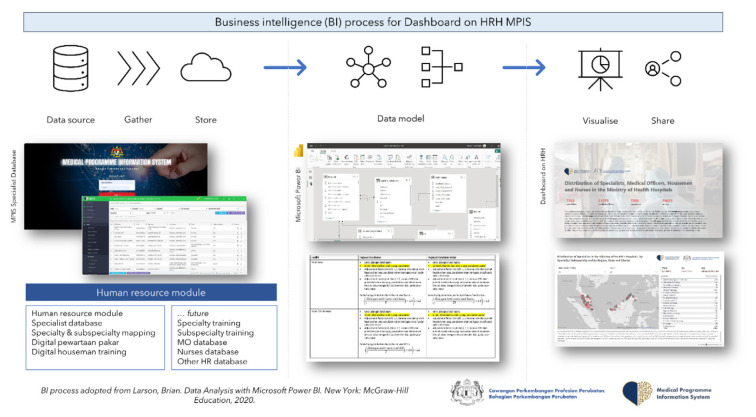
BI process of the specialist database module in the MPIS

Specialists Database Module in the MPIS has become an important element in the business intelligence (BI) process to monitor the distribution of medical specialists within the MOH Malaysia hospitals. Through a more defined database on specialists’ location, a more structured relational databases could be established with other relevant databases such as facility profile, workload and facility utilisation. A specific data modelling could be also developed, to provide a better insight on specialist distribution and its relationship with parameters mentioned above such as facilities, workload and population profile. Microsoft Excel Power Query was used primarily to achieve these and Microsoft Power BI was used as a platform for data visualisation. The BI process is demonstrated in Figure 1, adopted from Larson (5). Specialist database in all localities is updated from to time to time through a focal person appointed at each hospital facility. Users training were conducted by regions to assist these focal persons to understand and familiarise themselves with the system.

### Distribution of Medical Specialists

At the time of writing this article, there were 9,726 specialists in all 29 specialty areas serving the MOH Malaysia’s facilities nationwide including in the public health sector (public health and family medicine). There were 8,146 specialists serving the hospitals. There were 2,020 specialists trained in subspecialty areas listed in NSR (23%), 748 specialists trained in subspecialty areas not listed in NSR (9%) and 310 specialists trained in various areas of interest (4%). There were 1,477 specialists (17%) currently undergoing training in various subspecialty areas and areas of interest. The distribution of specialists by subspecialty and areas of interest mentioned above does not include public health and family medicine specialists.

There are many ways to analyse and summarise data on specialists’ location collected through MPIS. Descriptive analysis is one way and on top of that a more defined modelling can also be used to provide some insight on specialists’ distribution in the MOH Malaysia’s hospitals. Needs versus supply ratio by geographical localities is proposed to visualise status of ‘current distribution or supply’ against ‘quantified needs’ of specialists in each location. To demonstrate the use of such ratio in this paper, Malaysia was divided into six regions; region 1 to region 6. In the context of this paper, quantified needs means pre-defined targeted number of specialists to be achieved by 2030 by each speciality area and by each locality, or in this case, region. Current distribution or supply means number of resident specialists currently posted to the MOH Malaysia’s hospitals within each region. Needs versus supply ratios were calculated as follows:


$$\text{Needs versus supply ratio}=\frac{\text{Quantified }{\text{needs}}_{\text{(by year 2030)}}}{\text{Current }{\text{supply}}_{\text{(current yerar 2024)}}}$$

**Figure 2:**
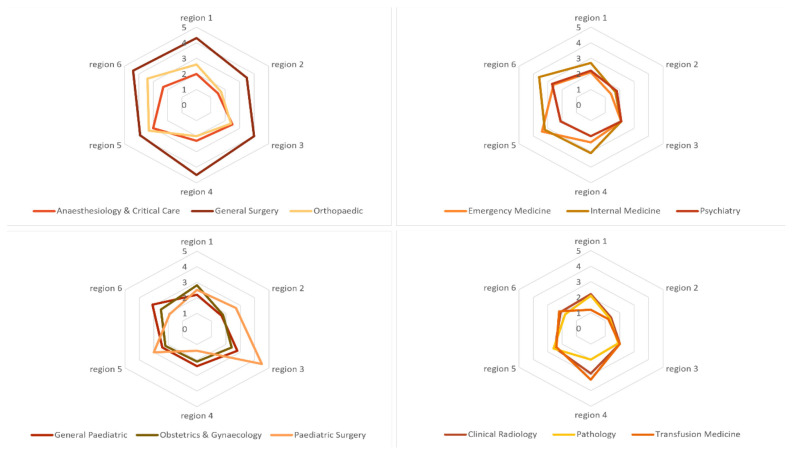
Needs versus supply ratio of selected specialty areas, by regions in Malaysia

A ratio of ‘1’ means the number of quantified needs is equivalent to the number of supply, which signifies the current supply of specialists has met the needs of specialist in a particular specialty for a given locality. Whilst a ratio of ‘2’ for example, means the quantified needs is 2 times the number of current supply of specialists, thus the need to bridge the gap. A ratio of less than ‘1’ means the supply is more than the quantified needs. Needs are quantified using adjusted population-based targets of specialists in all 25 basic specialty areas published in previous editorial in this journal (6). Number of specialists needed by 2030 in each specialty were obtained using these population-based targets against the projected population count in Malaysia by 2030. The same principle in the previous editorial applies, where at least 70% of these estimated number of specialists needed shall serve the public sector considering and assuming the disease burden profile and division between public-private sector remain the same in the future. The estimated numbers of specialists needed in Malaysia by 2030 in each specialty were then redistributed to each region based on workload, facilities and geographical profile. Several quantifiable factors were identified for the redistribution and adjustment namely population count, size of each region, outpatient attendances in both hospital and primary care settings, hospital admissions, deliveries, case-mix index, size of specialist and non-specialist hospitals and number of health clinics including more advance categories of clinics (also known as KK1 and KK2 clinics) providing more complex level of care. Each of these factors was weighted based on expert opinion. Each region was given score using these quantifiable factors adjusted by their respective weight (adjusted score). The estimated numbers of specialists needed by 2030 in each specialty were apportioned using the adjusted score for each region. Based on estimated quantified needs as described above and the current supply of specialists for each specialty area in each region, the needs versus supply ratio can now be calculated. Spider web diagram was used to visualise and compare the calculated ratio for each region (Figure 2). A few specialty areas were selected for visualisation and discussion in this paper.

Ideally, the web for each specialty on the diagram shall be located centrally nearing the ratio of ‘1’ and equally distributed in all regions. It is noted that in some specialties, the ratio is relatively higher compared to other specialties and in some regions, the ratio is observed to be higher compared to other regions. Number of specialists in certain specialties may be perceived to be high in certain parts of the country, but if we were to compare that with estimated needs in each locality, current supply of specialists in any specialty may not necessarily have met the estimated needs for that region. The quantified needs may be higher in certain regions due to several reasons such as higher workload, higher referrals to a particular region, complexity of cases, higher population density, availability of more advance subspecialty services or facilities and so forth. High and unequal distribution of such ratio shall be investigated and further analysed. While it is acknowledged that distribution of specialists shall be even in all localities, several factors could have contributed to the existing pattern in spider web diagram. Higher attrition rate especially to the private sector and low production of specialists through the existing local specialist training programme are among important factors for the high ratio. Readiness of health facilities in certain regions such as inadequate operation theatre, radiological or imaging facilities and other medical equipment are among factors to explain why specialists could not be posted to certain localities. Inadequate clinical support staff such as nurses, allied health professionals and other services such as physiotherapy may also affect the expansion of specialist services in certain localities. Willingness to be posted as resident specialists in localities considered to be unpopular is another important determining factor. Short-term and long-term measures to address the high and unequal distribution of such ratio shall be specified according to these factors. Availability of post is often made through historical arrangement and not necessarily mirror the actual needs. This issue is not exclusive to the Malaysian healthcare system, but it is also an acknowledged issue within the National Health Service (NHS) in the UK (7). The NHS is reforming its education and training to address geographical inequity. Such reform can be considered as part of the short-term and long-term measures to be adopted in our system.

**Figure 3:**
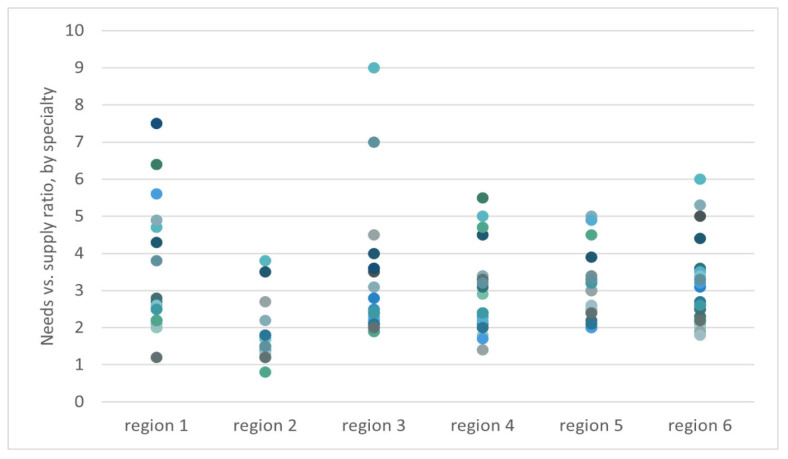
Needs versus supply of all specialty areas, by regions in Malaysia

Plotting the needs versus supply ratio of all specialty areas by each region, as shown in Figure 3, revealed an interesting ‘meteor-shower-like’ pattern. Most specialty areas in all regions are moving towards lower ratio and only minimal number of specialty areas are located at the extreme end of ‘tail’ of the ‘meteor shower’. Such an outlier ratio shall be addressed accordingly. Median of the ratio is 2.4.

## Conclusion

It is essential to acknowledge the importance of a robust system or mechanism to specifically map the distribution of existing medical specialists within the MOH Malaysia’s hospitals, given the dynamics of their locations and demands. Master list of specialty and subspecialty framework and the Specialist Database Module in the MPIS have been used as tools to establish relational databases and data visualisation for such mapping purposes. Such tools shall be improvised for sustainable use in the future.

## Appendix

[Table t1-01mjms3103_ed]

**Table t1-01mjms3103_ed:** Master list of specialty, subspecialty areas and areas of interest

Broad category	Specialty	Category	Clinical area
Medical related	Emergency Medicine	Subspecialty Area of Interest	Paediatric Emergency Medicine Clinical Toxicology Emergency Critical Care Emergency Trauma Care Pre-hospital Care and Disaster
	Internal Medicine	Subspecialty	Cardiology Clinical Haematology Dermatology Endocrinology Gastroenterology & Hepatology Geriatric Medicine Infectious Diseases Intensive Care Medicine Medical Oncology Nephrology Neurology Palliative Medicine Respiratory Medicine Rheumatology Acute Internal Medicine
	Oncology	Subspecialty	Clinical Oncology Radiation Oncology
	Psychiatry	Subspecialty	Addiction Psychiatry Child and Adolescent Psychiatry Community and Rehabilitation Psychiatry Consultation-Liaison Psychiatry Forensic Psychiatry Geriatric Psychiatry Neuropsychiatry
	Rehabilitation Medicine	Area of interest	Amputee Rehabilitation Cancer Rehabilitation Cardiac Rehabilitation Community Rehabilitation Critical Care Rehabilitation Geriatric Rehabilitation Musculoskeletal Rehabilitation Neuro-Rehabilitation Paediatric Rehabilitation Pulmonary Rehabilitation
Surgical-and-anaesthesia related	Sport Medicine		
	Anaesthesiology & Critical Care	Subspecialty	Intensive Care Cardiothoracic Anaesthesiology & Perfusion Liver Anaesthesia Neuro-anaesthesia & Trauma Obstetric Anaesthesia Paediatric Anaesthesia Pain Management Regional Anaesthesia Vascular Anaesthesia
	Cardiothoracic Surgery		
	General Surgery	Subspecialty	Breast and/Endocrine Surgery Colorectal Surgery Hepatobiliary Surgery Thoracic Surgery Upper Gastrointestinal Tract Surgery Vascular Surgery Trauma Surgery
		Area of interest	Breast and/Endocrine Surgery (GSWI) Colorectal Surgery (GSWI) Hepatobiliary Surgery (GSWI)
	Neurosurgery	Area of interest	Functional Surgery Neurovascular Paediatric Neurosurgery Skull Base Surgery Spine Surgery
	Ophthalmology	Subspecialty	Comprehensive Ophthalmology Cornea Glaucoma Medical Retina Neuro Ophthalmology Oculoplastic Surgery Paediatric Ophthalmology Public Health Ophthalmology Vitreoretinal
	Orthopaedic	Subspecialty	Advanced Musculoskeletal Trauma Arthroplasty Arthroscopy & Sports Surgery Foot & Ankle Orthopaedics Oncology Paediatric Orthopaedics Spine Surgery Upper Limb & Microsurgery
	Otorhinolaryngology	Subspecialty	Head & Neck Surgery Otology/Skull Based Surgery Paediatric Otorhinolaryngology Rhinology
		Area of interest	Audiovestibular
	Paediatric Surgery	Area of interest	Paediatric Hepatobiliary Surgery and Liver Transplantation Paediatric Reconstructive Urology Paediatric Surgical Oncology
	Plastic Surgery Urology		
Mother-and-child related	General Paediatric	Subspecialty	Adolescent Medicine Clinical Genetics Developmental Paediatrics Neonatology Paediatric Cardiology Paediatric Dermatology Paediatric Endocrinology Paediatric Gastroenterology Paediatric Haematology & Oncology Paediatric Infectious Disease Paediatric Intensive Care Paediatric Nephrology Paediatric Neurology Paediatric Respiratory Medicine Paediatric Rheumatology Paediatrics and child health Paediatric Emergency Medicine Paediatric Neuro-disability and Rehabilitation Medicine Paediatric Palliative Medicine
		Area of interest	Paediatric Cardiology Paediatric Haematology Paediatric Intensive Care Paediatric Nephrology Paediatric Oncology
	Obstetrics & Gynaecology	Subspecialty	Gynae-Oncology Maternal Fetal Medicine Reproductive Medicine Uro-gynaecology
Pathology-and-lab related	Anatomical Pathology	Area of Interest	Breast & Endocrine Pathology Cytopathology Dermatopathology Gastrointestinal Tract & Hepatobiliary Pathology Gynaecology Pathology Head & Neck Pathology Lung & Thoracic Pathology Lymphoreticular Pathology Molecular Pathology Neuropathology & Neuromuscular Ocular Pathology Paediatric Pathology Perinatal Pathology Renal Pathology Soft tissue and Bone Pathology Uropathology
	Chemical Pathology	Subspecialty	Chemical pathology (metabolic medicine)
		Area of Interest	Endocrine/Metabolic Molecular Paediatric (Inborn Errors of Metabolism) Protein/Proteomic Toxicology
	Forensic Pathology	Area of Interest	Clinical Forensic Medicine Forensic Anthropology Forensic Cardiopathology Forensic Histopathology Forensic Human Identification Forensic Neuropathology Forensic Paediatric Pathology
	General Pathology Genetic Pathology		
	Haematology	Area of Interest	Bone Marrow Cytogenetics (Genetics in Haematology Disorder) Haemostasis & Thrombosis Molecular Haemato-oncology Paediatric Haematology Red Cell Enzyme and Membrane Disorders Stem Cell Transplantation Thalassaemia Haemoglobinopathy
	Medical Microbiology	Area of Interest	Cardiac & Respiratory Infection Herpesviridae and Hepatitis HIV Drug Resistance Infection Control Molecular Bacteriology Molecular Virology Mucormycosis Mycology in Immuno-compromised Patient Paediatric Infectious Disease Parasitic Infection Sexually Transmitted Infection Transplant Virology Tuberculosis & Leprosy
	Transfusion Medicine	Area of Interest	Apheresis Cellular Therapy Clinical Transfusion Donation & Donor behaviour Good Manufacturing Practice Hemovigilance Histocompatibility & Immunogenetic Immuno-haematology & Clinical Transfusion Immunohaematology (red cell/white blood cell/platelet) Patient Blood Management Quality Management in Transfusion Medicine Regenerative Medicine (Platelet Rich Plasma) Surveillance Transfusion Microbiology Transplant Immunology
Radiology related	Clinical Radiology	Subspecialty	Breast Radiology Cardiac Radiology Gastrohepatobiliary Radiology Genitouroradiology Head & Neck Radiology Interventional Radiology Musculoskeletal Radiology Neuroradiology Oncoradiology Paediatric Radiology Thoracic Radiology
		Area of Interest	Breast Radiology Cardiac Radiology Forensic Radiology Head & Neck Radiology Neuroradiology Thoracic Radiology
	Nuclear Medicine	Area of Interest	Nuclear Cardiology Nuclear Neurology Nuclear Oncology Paediatric Nuclear Medicine Theranostic
Community related	Family Medicine	Subspecialty	Addiction Medicine in Primary Care Adolescent Health in Primary Care Child Health in Primary Care Communicable Disease in Primary Care Geriatric in Primary Care Mental Health in Primary Care Non-Communicable Disease in Primary Care Palliative Medicine in Primary Care Rehabilitation in Primary Care Sexual and Reproductive Health in Primary Care
		Area of Interest	Clinical Epidemiology and Statistics Dermatology, FM Health Informatics Respiratory Medicine, FM Travellers’ Health
	Public Health	Subspecialty	Communicable Disease Epidemiology Environmental Health Family Health Health Management Military Medicine Non-Communicable Disease Epidemiology Occupational Health
Dental-related specialities	Dental Public Health	Subspecialty	Community Oral Health Oral Health Epidemiology Oral Health Service Management
	Endodontics Forensic Odontology Oral and Maxillofacial Radiology		
	Oral and Maxillofacial Surgery	Subspecialty	Dentofacial Deformity Surgery Craniomaxillofacial Trauma and Bone Surgery Maxillofacial Oncology and Reconstructive Surgery,
	Oral Pathology and Oral Medicine/Oral Medicine Orthodontic		
	Paediatric Dentistry	Subspecialty	Paediatric Orofacial Anomalies / Malformation Paediatric Prosthodontics
	Periodontology Prosthodontics	Subspecialty	Management of Peri-implant Disease
	Restorative Dentistry	Subspecialty	Geriatric Prosthodontics Maxillofacial Prosthodontics Regenerative Endodontics
	Special Care Dentistry		

Notes: ________ Underlined clinical areas are those listed as registrable specialty or subspecialty areas in the National Specialist Register (NSR) under the Malaysian Medical Council; Double-underlined clinical areas are those listed as registrable specialty areas under the Malaysian Dental Council; GSWI = general surgeon with interest
